# LSD1-ERRα complex requires NRF1 to positively regulate transcription and cell invasion

**DOI:** 10.1038/s41598-018-27676-8

**Published:** 2018-07-03

**Authors:** Ling Zhang, Julie Carnesecchi, Catherine Cerutti, Violaine Tribollet, Séverine Périan, Christelle Forcet, Jiemin Wong, Jean-Marc Vanacker

**Affiliations:** 1 Shanghai Key Laboratory of Regulatory Biology, Institute of Biomedical Sciences, School of Life Sciences, East China Normal University, Shanghai, 200241 China; 2 Institut de Génomique Fonctionnelle de Lyon, Université de Lyon, Université Lyon 1, CNRS UMR5242, Ecole Normale Supérieure de Lyon, 32-34 Avenue Tony Garnier, F-69007 Lyon, France; 3 Present Address: Department of Developmental Biology, Centre for Organismal Studies (COS), Heidelberg, University of Heidelberg, D-69120 Heidelberg, Germany

## Abstract

Lysine-specific demethylase 1 (LSD1) exerts dual effects on histone H3, promoting transcriptional repression via Lys4 (H3K4) demethylation or transcriptional activation through Lys9 (H3K9) demethylation. These activities are often exerted at transcriptional start sites (TSSs) and depend on the type of enhancer-bound transcription factor (TFs) with which LSD1 interacts. In particular, the Estrogen-Receptor Related α (ERRα) TF interacts with LSD1 and switches its activities toward H3K9 demethylation, resulting in transcriptional activation of a set of common target genes. However, how are the LSD1-TF and, in particular LSD1-ERRα, complexes determined to act at TSSs is not understood. Here we show that promoter-bound nuclear respiratory factor 1 (NRF1), but not ERRα, is essential to LSD1 recruitment at the TSSs of positive LSD1-ERRα targets. In contrast to ERRα, NRF1 does not impact on the nature of LSD1 enzymatic activity. We propose a three factor model, in which the LSD1 histone modifier requires a TSS tethering factor (NRF1) as well as an activity inducer (ERRα) to transcriptionally activate common targets. The relevance of this common network is illustrated by functional data, showing that all three factors are required for cell invasion in an MMP1 (Matrix MetalloProtease 1)-dependent manner, the expression of which is regulated by NRF1/LSD1/ERRα-mediated H3K9me2 demethylation.

## Introduction

Positive regulation of gene expression is achieved by transcription factors (TFs) that bind to promoters and/or to discrete genomic sites (enhancers) located more or less distal, upstream or downstream, from the transcriptional start sites (TSSs) of the genes they regulate^1–3^. These TFs recruit, amongst others, co-activator proteins that modify histone tails. The latter activities are often exerted at the TSSs of the targeted genes, and eventually lead to the induction of consistent transcriptional programs that regulate distinct physiopathological traits. How are enhancer-promoter dialogs finely regulated and, in particular, what are the determinants at the TSSs that instruct TF-Co-A complexes where to act are unclear.

Lysine Specific Demethylase 1 (LSD1; KDM1A) is an enzyme that demethylates histone and non-histone substrates and exerts dual activities on histone H3 (H3)^4–11^. By demethylating mono- and dimethyl Lys4 on histone H3 (H3K4), LSD1 induces transcriptional repression. In contrast, when demethylating mono- and dimethyl H3K9, LSD1 acts as a transcriptional co-activator, promoting the expression of its target genes. LSD1 can be recruited by various TFs at their cognate response element, including in enhancer regions, and can exert its activities at enhancers and/or TSSs^12–16^. The Nuclear Respiratory Factor 1 (NRF1) TF has been suggested as a key factor to tether LSD1 to TSSs and, together with LSD1, induces the expression of genes involved in oxidative phosphorylation in white adipose tissue^17,18^. However, the relationships of the LSD1-NRF1 complex with upstream bound TFs are not understood.

The Estrogen-Related Receptor α (ERRα) is an orphan member of the nuclear receptor TF family^19^ that regulates various physiopathological features. ERRα is largely involved in the regulation of cellular metabolism, an activity that strongly depends on its interactions with the PGC-1 family of co-activators^20,21^. ERRα is also highly expressed in aggressive tumors and promotes different processes linked to cancer progression, such as resistance to hypoxia, angiogenesis, metabolic switch toward aerobic glycolysis, orientated cell migration and extracellular matrix invasion^22–27^. Our recent data suggest a connection between LSD1 and the pro-migratory activities of ERRα, with both factors inducing a common transcriptional program that is highly enriched in genes involved in the control of cell invasion^15^. Positive co-targets are regulated via the demethylation of H3K9me2 at their TSS, an activity that is exerted by LSD1 in an ERRα-controlled manner and that reflects *in vitro* assays showing that, in the presence of ERRα, LSD1 demethylates H3K9me2. However, the determinants that specifically position the enhancer-associated LSD1-ERRα complex to exert such activities at the TSSs are unknown.

In the present report, we show that the TSSs of targets that are positively regulated by both LSD1 and ERRα (hereafter referred to as LSD1-ERRα positive targets) are selectively enriched in NRF1 binding motifs. Consistently, these positive targets are regulated by NRF1 via its capacity to recruit LSD1, eventually leading to local H3K9me2 demethylation. Furthermore, LSD1 activity on these genes depends on both ERRα and NRF1. As LSD1 and ERRα, NRF1 is required for cell invasion through its ability to induce the expression of the MMP1 metalloprotease.

## Results

### LSD1-ERRα responsive promoters are enriched in NRF1 motifs

**Figure 1:**
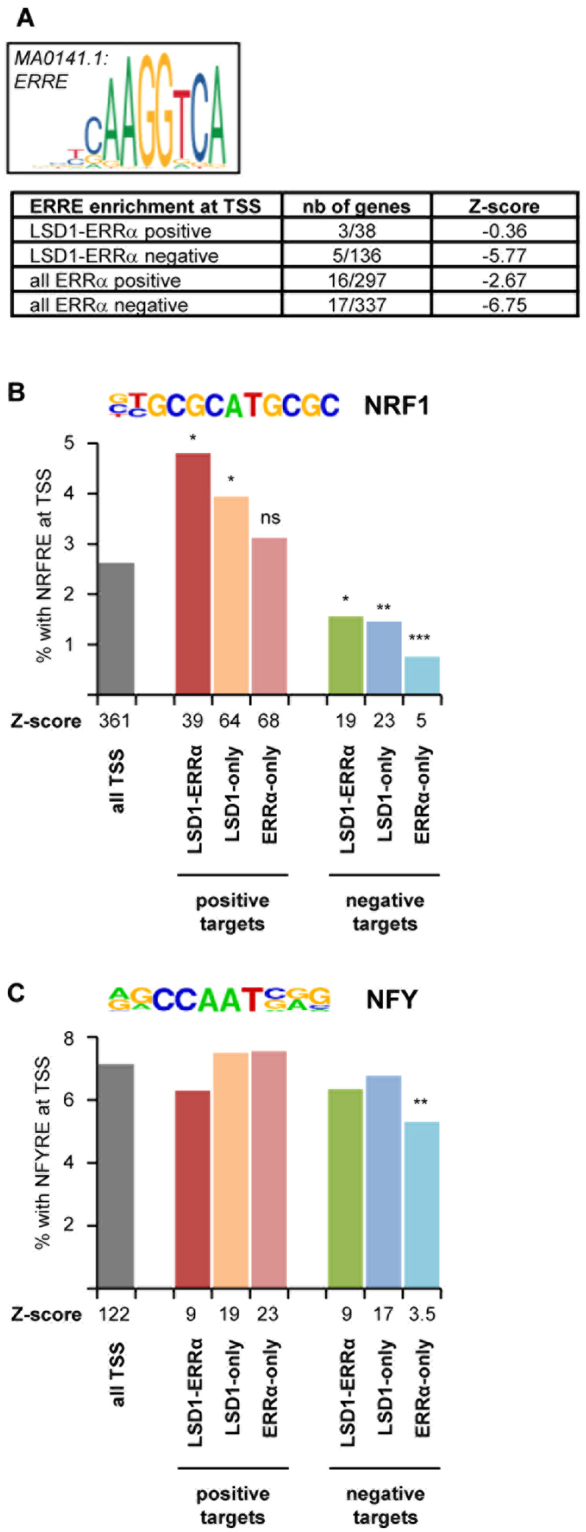
Common LSD1-ERRα positive targets are enriched in NRF1 motif in their TSS. (**A)** Enrichment in ERRα response element (ERRE; JASPAR matrix displayed) in the different categories of LSD1 and ERRα responsive genes expressed as Z-scores. Proportion of genes displaying ERRE motif is also indicated. (**B,C)** Percent of sequences displaying at least 90% similarity to NRF1 motif (**B**) NFY motif (**C**) in their TSS. NRF1 and NFY HOMER core motifs are indicated. The Z-score for motif enrichment (>10) *vs* random sequences is given below the x-axis. Significance was calculated relative to “all TSS” with *p < 0.05, **p < 0.01, ***p < 0.005, ns: nonsignificant. Note that negative targets in (**B)** display a significant under-representation. Values in (**c)** are nonsignificant except where indicated.

To analyse the mechanisms through which the LSD1-ERRα complex is recruited at the TSS of their common target genes (defined in MDA-MB231 cells in our previous work^15^), we performed *in silico* analyses of these TSSs (defined as −150/+50 relative to +1 nucleotide of transcribed mRNA). We first found that the ERRα response element (ERRE) itself is not enriched at TSSs, neither of (positive or negative) common LSD1-ERRα targets nor at all-ERRα (*i*.*e*. responding or not to LSD1) targets (Fig. 1A). Indeed, the negative Z-scores obtained with this motif betrayed a trend toward under-representation relative to all possible TSSs. This suggests that ERRα does not directly bind the TSS of its target genes and that other factor(s) is involved in its recruitment. We next focused on NRF1 since it has been suggested as an LSD1-tethering factor^17,18^. In a first stringent screen, we found an enrichment in putative NRFRE when comparing all possible TSSs to random DNA (*i*.*e*. non-TSS) sequences (Fig. 1B), as expected for a TF that preferentially binds in promoter-proximal regions. Interestingly, this enrichment, which was also detected at the TSSs of positive LSD1 and/or ERRα targets, was largely increased compared to all possible TSSs for LSD1 positive targets, in particular, common LSD1-ERRα ones but not for ERRα-only targets. A further screen using less stringent conditions also indicated that the TSSs of LSD1-ERRα positive targets were particularly enriched in putative NRFRE, although LSD1-only as well as ERRα-only targets were also enriched (Supplementary Fig. S1a). In contrast, this enrichment was not observed on negative LSD1 and/or ERRα targets as compared to all possible TSSs (Fig. 1B and Supplementary Fig. S1a). As a specificity control, we analysed the same sub-groups of genes for a possible enrichment in the binding motif for NFY, an unrelated promoter-proximal-binding TF (Fig. 1C). As expected, enrichment for NFYRE was clearly detected for all possible TSSs, and appeared of variable importance across other TSS groups. However, no over-representation of NFYRE was found for the LSD1 and/or ERRα targets compared to all possible TSSs.

### NRF1 regulates LSD1-ERRα targets

**Figure 2:**
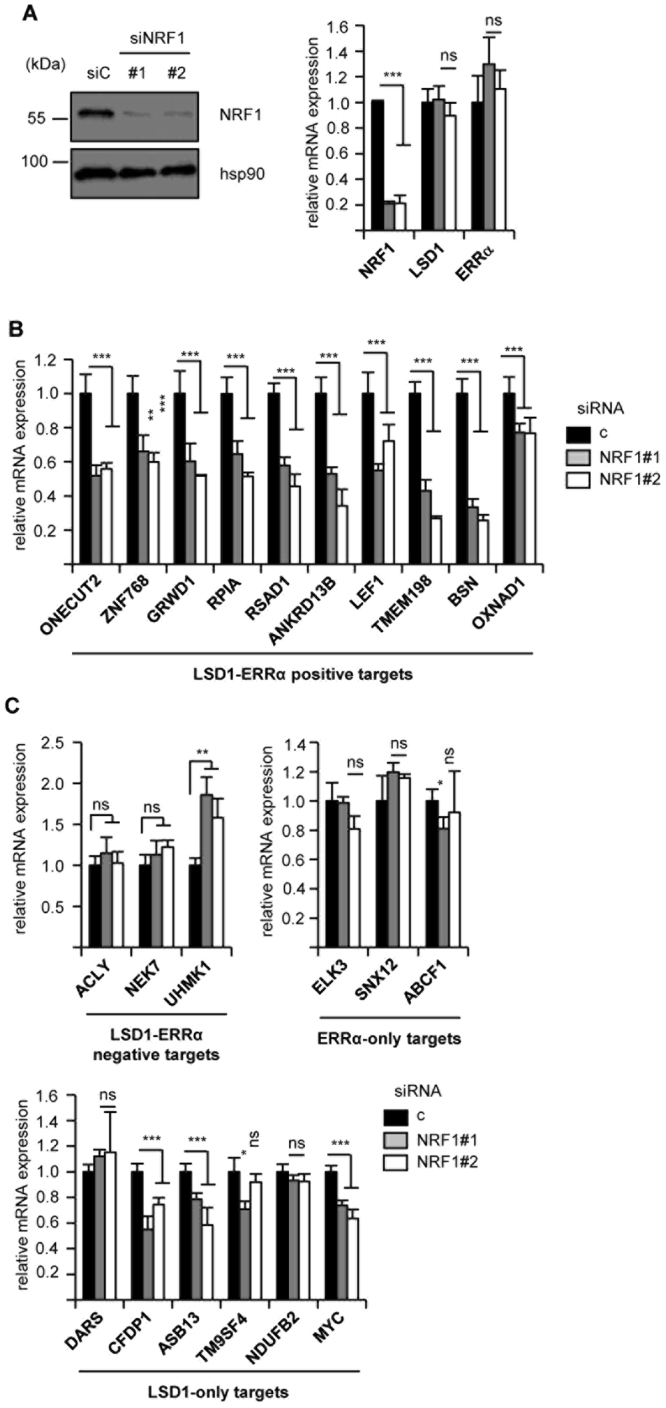
Regulation of ERRα-LSD1 targets by NRF1. **(A)** Efficiency of siRNA directed at NRF1. Expression of NRF1 protein (analysed by Western blot, left panel) or of the indicated mRNA (analysed by RT-qPCR, right panel) after transfection of the NRF1 siRNA in MDA-MB231 cells. (**B)** Expression of ERRα-LSD1 common positive targets after transfection with siRNA directed against NRF1, analysed by RT-qPCR. (**C)** Expression of LSD1-ERRα negative (upper left panel), ERRα-only (upper right panel) or LSD1-only (lower panel) targets after siNRF1 transfection. Data are expressed as mean +/− sem of three independent experiments performed in triplicate. Significance are shown relative to control: *p < 0.05, **p < 0.01, ***p < 0.005, ns: nonsignificant.

Altogether this suggests that the LSD1-ERRα complex could be recruited at the TSS of their positive targets via NRF1, a hypothesis that we next investigated. We focused on a set of 10 LSD1-ERRα targets that we previously defined as positively responding to both factors^5^. A bio-informatic screen of the promoters of these genes indicated that all comprised a putative NRFRE (>84% similarity to consensus) in the vicinity of the TSS (Supplementary Fig. S1b). We reasoned that the expression of LSD1-ERRα positive target should also depend on the presence of NRF1. Inactivation of this factor by two different siRNA (validated at the protein and mRNA level on Fig. 2A) resulted in decreased expression of these LSD1-ERRα positive targets (10/10; Fig. 2B) in MDA-MB231 cells. In contrast, NRF1 regulation of LSD1-ERRα negative targets was only observed in 1 out of 3 tested genes (Fig. 2C). 3 out of 6 LSD1-only (positive) genes and 0 out of 3 ERRα-only genes were found as NRF1-responsive. These experimental data are consistent with the *in silico* analysis above.

**Figure 3:**
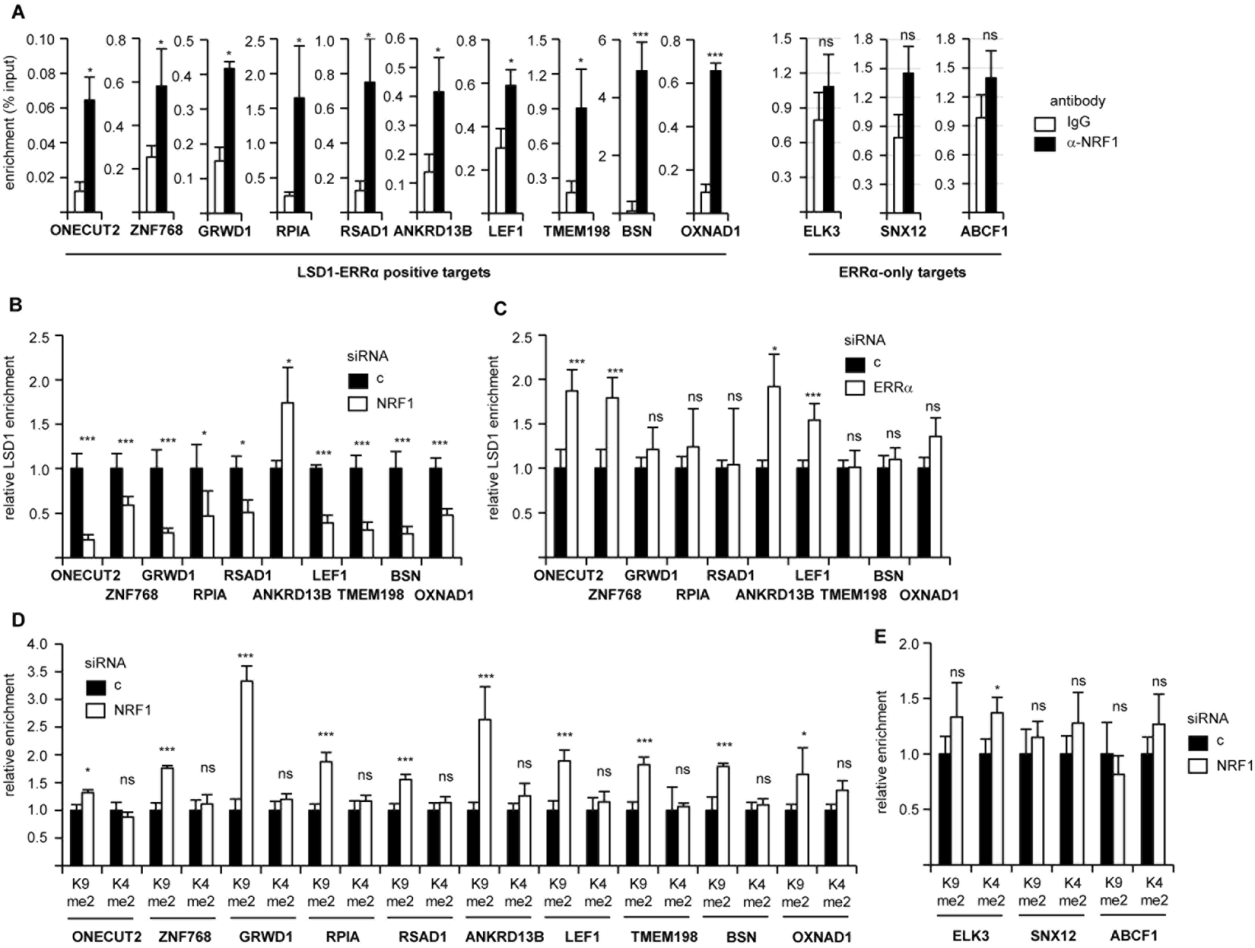
NRF1 promotes LSD1 recruitment at target TSS. (**A)** ChIP experiments using anti-NRF1 or IgG. Percent enrichments relative to input were measured by qPCR, amplifying a region encompassing the TSS of the indicated genes. (**B,C)** ChIP experiments using anti-LSD1 on chromatin from MDA-MB231 cells treated siRNA against NRF1 (**B**) or ERRα (**C**). Enrichments are shown relative to control (c) conditions, amplifying the same genomic regions as above. (**D)** ChIP experiments using anti-H3K4me2 or anti-H3K9me2 antibodies on chromatin from MDA-MB231 cells treated by siNRF1. Enrichments are shown relative to H3 ChIP and to control conditions, amplifying the same genomic regions as above. (**E)** ChIP experiments as in (**D**), targeting the TSS of genes that only respond to ERRα, not LSD1. Data are expressed as mean +/− sem of three independent experiments performed in duplicate. Significance are shown relative to control: *p < 0.05, **p < 0.01, ***p < 0.005, ns: nonsignificant.

Our hypothesis predicts that NRF1 would be recruited at the TSSs of LSD1-ERRα positive targets. Investigating this possibility by Chromatin ImmunoPrecipitation (ChIP)-qPCR, we found a significant enrichment of NRF1 protein on all considered TSSs, but not on those of ERRα-only genes (Fig. 3A). We next analysed whether NRF1 binding at the TSSs is required for LSD1 recruitment in these regions. To this end, NRF1 was inactivated by siRNA and LSD1 was immunoprecipitated from the resulting extracts. In 9 out of 10 cases, this treatment resulted in a strong decrease in LSD1 recruitment, relative to control conditions (Fig. 3B). In contrast, siRNA-mediated ERRα depletion had no effect on LSD1 presence at the TSS (Fig. 3C), indicating that NRF1, but not ERRα, is critical for LSD1 positioning at the TSS. As such, depletion of NRF1 should result in local chromatin modifications that are similar to those observed when depleting LSD1, *i*.*e*. increased H3K9me2. As indicating by ChIP-qPCR experiments, NRF1 inactivation consistently resulted in enhanced H3K9me2 deposition (indicating transcriptional repression) at the TSS of all tested LSD1-ERRα positive genes (Fig. 3D). In contrast, the level of the H3K4me2 (reflecting transcriptional activation) did not vary upon NRF1 depletion. Furthermore, this treatment did not result in significant variations in H3K9me2 or H3K4me2 levels on the TSS of NRF1 unresponsive genes (Fig. 3E). Altogether this shows that NRF1 mediates LSD1 correct positioning at the TSS of its target genes, resulting in transcriptional activation through LSD1-induced, ERRα-controlled H3K9me2 demethylation.

**Figure 4:**
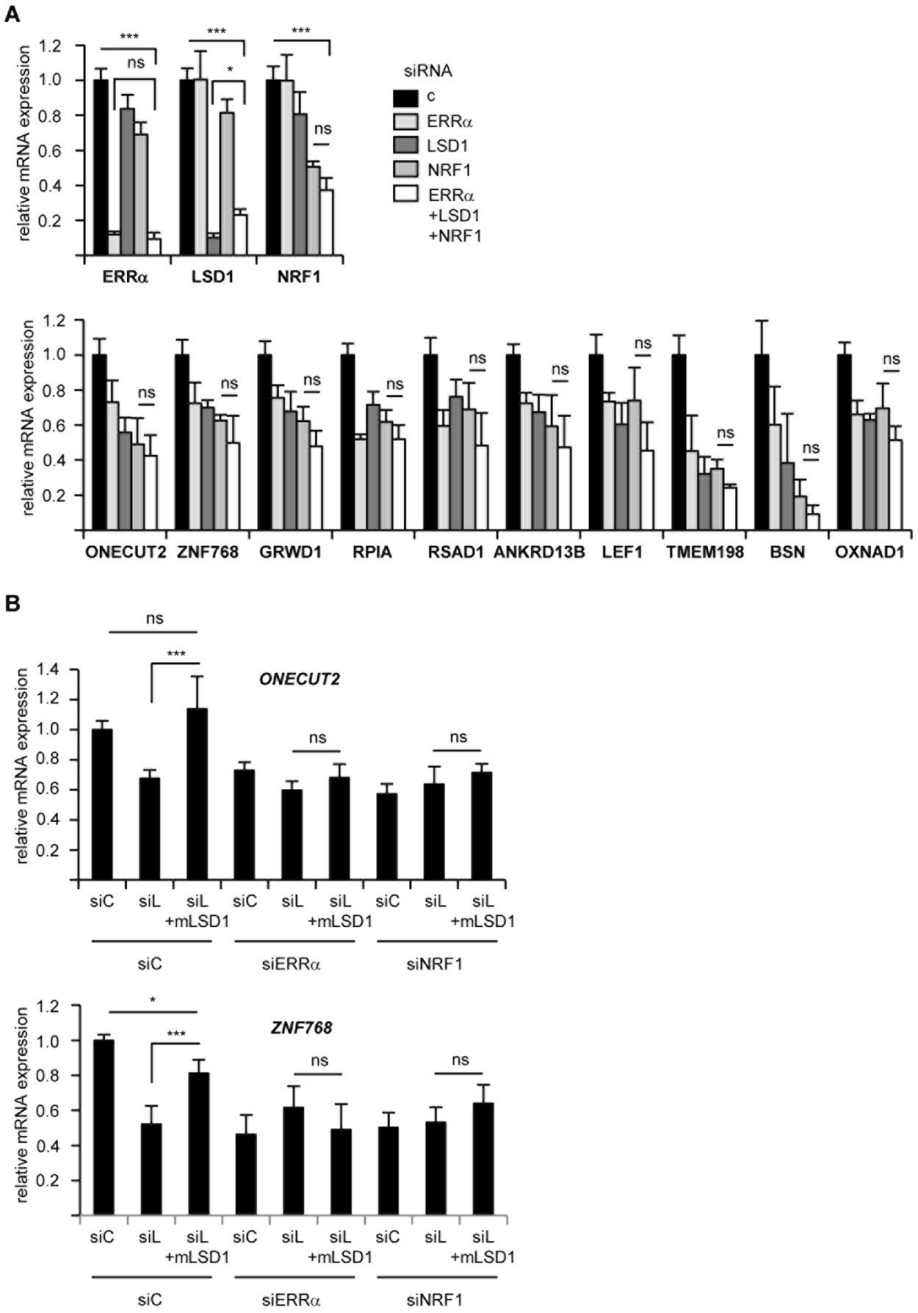
LSD1 activity depends on both NRF1 and ERRα. (**A)** Expression of the indicated genes after single or triple knock down in MDA-MB231 cells. Expression of NRF1, LSD1 and ERRα (displayed on the upper panel) shows similar inactivation under single and triple KD conditions. (**B)** Expression of LSD1-ERRα positive targets in HEK293T cells under the indicated conditions. Where indicated, cells were transfected with mouse LSD1 encoding plasmid or empty plasmid (pCDNA3) as a control. Expression of two genes are displayed, see Supplementary Fig. S2 for that of other LSD1-ERRα, as well as control. Data are expressed as mean +/− sem of two independent experiments performed in triplicate. Significance are shown relative to control: *p < 0.05, **p < 0.01, ***p < 0.005, ns: nonsignificant.

This model predicts that all three factors act in the same pathway. If so, simultaneous depletion of all three factors should not result in an additive effect, as compared to inactivation of a single factor, an assumption that we next tested (Fig. 4A). Consistently, triple siRNA-mediated knock down (KD) did not further decrease target gene expression relative to single factor inactivation. Noteworthy, triple KD was as efficient as single one on each individual factor. The above model also implies that LSD1-mediated transcriptional activation requires both ERRα and NRF1, a possibility that we analysed through rescue experiments (Fig. 4B and Supplementary Fig. S2). For technical reasons, these experiments were performed in HEK293T cells and first show that the same gene set is deregulated upon siNRF1 as in MDA-MB231 (excluding TMEM198 which is not expressed in HEK293T cells). After treatment with siLSD1, cells were transfected or not with a mouse version of the demethylase (thus escaping the human-targeting siLSD1 treatment). Under these conditions, LSD1 replenishment normalized the expression level of the target genes. Incidentally, these expression levels did not rise above those observed under control conditions, despite the elevated expression of mouse LSD1, indicating the presence of endogenous limiting factors. Strikingly, when transfected in cells co-depleted from NRF1 or ERRα, mouse LSD1 was completely incapable of rescuing human LSD1 depletion. This indicates that both NRF1 and ERRα are required for LSD1 activity.

### NRF1 promotes cell invasion

**Figure 5:**
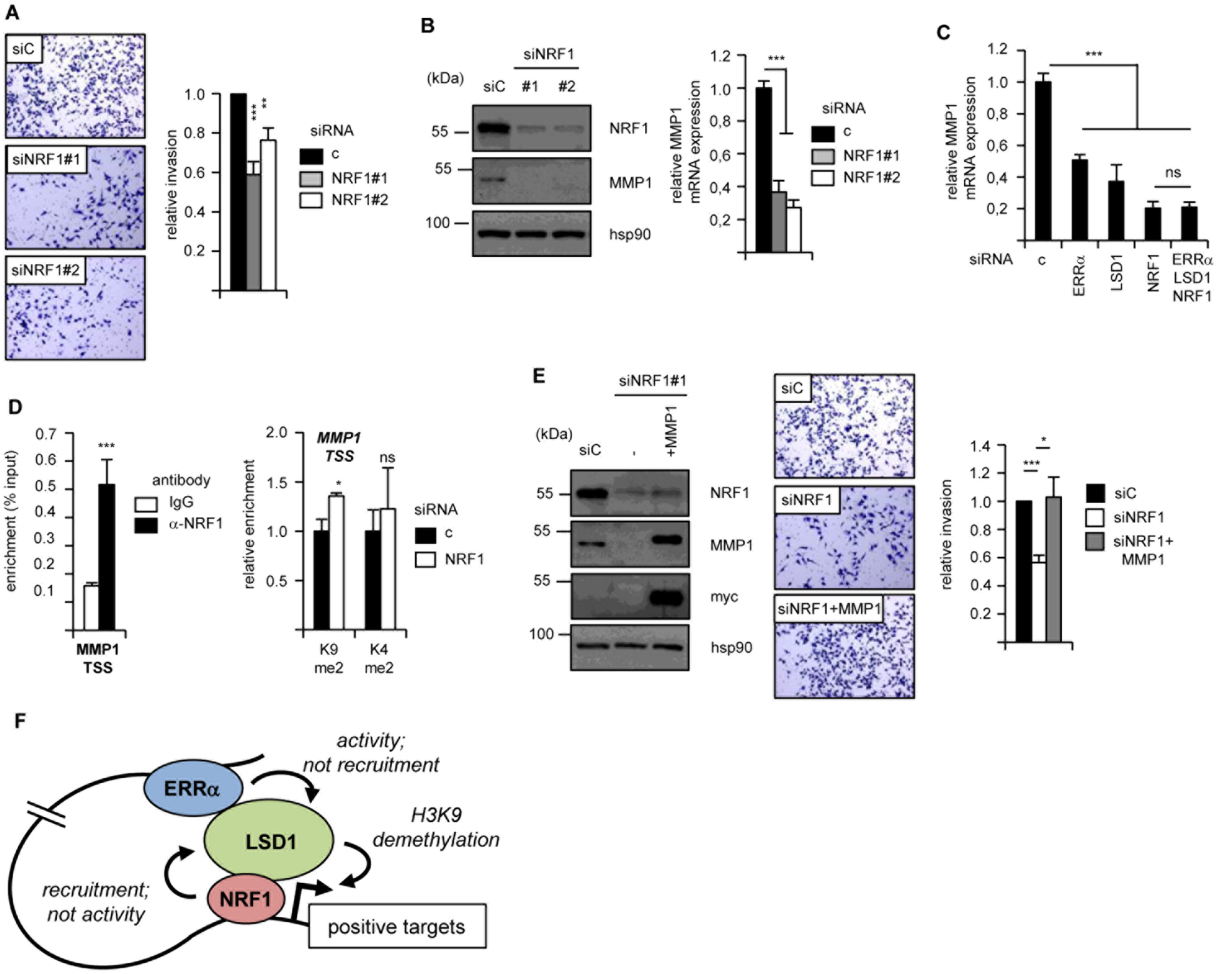
NRF1 promotes cell invasion in an MMP1-dependent manner. (**A)** Invasion assays of MDA-MB231 cells after treatment with siNRF1. Representative fields are displayed on the left. Quantifications were performed on three independent experiments and are shown relative to control conditions. Error bars represent sem. (**B**) Expression of MMP1 was analyzed by Western blot (left panel; Hsp90 is used as a loading control) or RT-qPCR (right panel; expression is relative to control) after siRNA-mediated inactivation of NRF1. (**C)** Expression of MMP1 mRNA under the indicated single or triple KD conditions. (**D)** Left panel: binding of NRF1 to the MMP1 TSS analyzed by ChIP and expressed as percent input. Right panel: ChIP experiments detecting H3K9me2 or H3K4me2 at the MMP1 TSS after siRNA-mediated NRF1 depletion. Data are relative to H3 ChIP and to control conditions. (E) Left panel: expression of the indicated proteins after siNRF1 treatment and MMP1 re-introduction. Note that the myc antibody only detects the transfected myc-tagged MMP1. Right panel: Invasion assays of MDA-MB231 cells after NRF1 depletion and re-introduction of MMP1, with quantifications relative to control. Data are expressed as mean +/− sem of three independent experiments performed in triplicate. Significance are shown relative to control: *p < 0.05, **p < 0.01, ***p < 0.005, ns: nonsignificant. (**F)** Schematic representation summarizing the functional LSD1-NRF1-ERRα interactions on positive target genes. NRF1, bound at the TSS, recruits LSD1 at this location, but does not impact on its enzymatic activity. ERRα interacts with LSD1 and switches its enzymatic activity but is not responsible for its recruitment to TSSs. All three factors regulate H3K9 demethylation at the TSS in a direct (LSD1) or indirect (NRF1, ERRα) manner, resulting in gene activation.

Our previous work has shown that LSD1-ERRα induce cell invasion in an MMP1 (Matrix Metallo-Protease 1)-dependent manner^15^. Since NRF1 is required for the transcriptional activities of this complex, we hypothesized that this promoter-binding TF would also be required for such cellular phenotype. siRNA-mediated NRF1 depletion indeed resulted in reduced cell invasion capacities as tested by extracellular matrix-dependent Boyden chamber assays (Fig. 5A). Interestingly the expression of MMP1 was decreased in the absence of NRF1, as evidenced at the protein and mRNA levels (Fig. 5B). Triple KD suggested that LSD1, NRF1 and ERRα act in the same molecular pathway (Fig. 5C). NRF1 was also recruited at the MMP1 TSS and its depletion resulted in increased H3K9me2, but not H3K4me2, deposition (Fig. 5D). Finally, re-introduction of MMP1 in NRF1-depleted cells resulted in normalized invasive capacities (Fig. 5E). Altogether this indicates that NRF1 regulates MMP1 and cell invasion in a manner that involves both LSD1 and ERRα.

## Discussion

Our previous work^15^ has shown that the ERRα TF can recruit the LSD1 histone demethylase at distally localized ERRα response elements (ERRE). LSD1 can act as a transcriptional repressor by demethylating H3K4me2 or as a transcriptional activator by demethylating H3K9me2^4–11^. *In vitro* experiments have shown that ERRα switches the activities of LSD1 from H3K4me2 to H3K9me2 demethylation^15^. In cells, this activity is exerted at the TSS of co-regulated genes, where both proteins were detected. However, how LSD1 is instructed to precisely act at TSSs is unknown. Indeed, positively responding TSSs are not particularly enriched in ERRE sequences, suggesting that ERRα is not involved in the tethering of LSD1 to the TSSs. Consistently, siRNA-mediated ERRα inactivation does not impact on LSD1 TSS recruitment. In contrast, as compared to all TSSs (>78,000 analyzed), those of ERRα-LSD1 positive, but not negative, targets are selectively enriched in binding motifs for NRF1, but not for NFY. Depending on the NRF1 motif used, only 5% to 40% of LSD1-ERRα positive TSS display a fully consensus NRFRE (Fig. 1A and Supplementary Fig. S1a). Yet this stringent screen opened the possibility that additional (LSD1-ERRα positive) TSSs may recruit NRF1 through a degenerated NRFRE. In support to this hypothesis, NRF1 was experimentally detected at the TSS of all 10 genes tested. Furthermore, depletion of this TF strongly reduced LSD1 recruitment at these regions. These results are in agreement with data published by others that indicate a strong association of LSD1 to NRF1 binding sites in TSS regions^17,18^. Consistently, siRNA-mediated NRF1 inactivation resulted in increased H3K9me2 deposition and reduced expression of LSD1-ERRα positive targets, whereas LSD1-ERRα negative- or ERRα-only targets were unaffected. We previously showed that NRF1, in contrast to ERRα, does not induce LSD1 to demethylate H3K9me2. Altogether, this allows to propose a three factor model (summarized on Fig. 5F) in which NRF1, but not ERRα, is involved in positioning LSD1 to TSS, whereas ERRα, but not NRF1, regulates LSD1 enzymatic activities. This model predicts that, in these LSD1-ERRα positive targets, LSD1 transcriptional activities requires both NRF1 and ERRα (although at different levels), a hypothesis that we confirm with rescue experiments. This model is likely to operate in various cell types, since all three factors regulate similar targets in HEK293T and MDA-MB231 cells. However, our initial RNA-Seq data^15^ indicate that many LSD1-dependent targets do not require ERRα, suggesting that other enhancer-bound TFs to be identified may impact on LSD1 activity.

The consequences of our mechanistic findings are illustrated by functional data. Previously, we found that the LSD1-ERRα complex regulates cell invasion by inducing the expression of the MMP1 metalloprotease. This is also the case for NRF1 which behaves similarly to LSD1 and ERRα by regulating MMP1 expression with similar outcomes. Both LSD1 and ERRα have been independently demonstrated as highly expressed in aggressive cancers where their elevated expression correlates with a poor prognosis, in particular in breast cancer^24,28–33^. Several traits of cancer progression, such as epithelial-to mesenchymal transition (EMT) and remodelling of extracellular matrix are promoted by these two factors^15,16,26,27^. It was recently shown that the expression of NRF1 also increases in cancers and constitutes a factor of poor prognosis^34^. Previous works had shown that NRF1, in link with members of the PGC-1 family of co-activators, promotes mitochondrial biogenesis and activity, including in cancers^35,36^, suggesting a role cancer metabolism. Our present data suggest that NRF1 is also involved in the promotion of cell invasion and extracellular matrix remodelling, which are hallmarks of aggressive cancers, through its functional interactions with LSD1 and ERRα.

## Methods

### Cells

MDA-MB231 and HEK293T cells were cultured in DMEM supplemented with 10% FCS, 10 U/ml penicillin and 10 µg/ml streptomycin. For siRNA transfection, 3.10^−5^ cells per ml were seeded in 6-well plate and 25 pmol/ml of total siRNA were transfected with INTERFERin (Polyplus Transfection) according to the manufacturer’s recommendations. Plasmid transfections were performed with JetPrime (Polyplus Transfection). siRNAs were from Invitrogen (ERRα, LSD1) or Eurogentec (NRF1) and sequences are shown on Supplementary Table 1. For invasion assays, 5.10^4^ cells were suspended in 200 µl DMEM/2% FCS and seeded on top of matrigel invasion chambers (Corning). Cells were allowed to migrate toward the lower chamber containing DMEM/10% FCS for 48 h. Matrigel was removed using cotton buds and cells were fixed 1 h with 4% formaldehyde, coloured with 0.1% crystal violet, and microphotographed. Cell-covered surfaces were quantified using ImageJ.

### Expression analyses

For western blot analysis, cells were lysed in NP40 or RIPA buffer supplemented with Protease Inhibitor Cocktail (Sigma-Aldrich). Proteins (25–50 µg) were resolved on 8 to 15% SDS-PAGE, blotted onto PVDF membrane (GE-Healthcare) and probed with specific antibodies after saturation. The antibodies (and their dilution) used in this study were: hsp90 (API-SPA-830, Enzo Life Sciences, 1/3,000), myc-tag (9E10, Covance, 1/2,000), MMP1 (AB6002, Millipore, 1/1,000), NRF1 (ab55744, Abcam, 1/3,000). Uncropped blots are presented on Supplementary Fig. S3.

Total RNAs were extracted by the guanidinium thiocyanate/phenol/chloroform method. 1 µg of RNA was converted to first strand cDNA using the RevertAid kit (ThermoScientific). Real time PCRs were performed in 96 well plates using the IQ SYBR Green Supermix (BioRad). Data were quantified by ΔΔ-Ct method and normalized to 36b4 expression. Sequences of the primers used in this study are shown on Supplementary Table S1. All genes studied here have been defined as belonging to the ascribed categories (positively or negatively responding to LSD1 and/or ERRα) in our previous publication^15^.

### ChIP

10 × 10^6^ cells were cross-linked with 1% formaldehyde and quenched for 5 min in 1 M Glycine. After centrifugation, cell pellets were resuspended in lysis buffer (1% SDS, 50 mM Tris-HCl pH8, 10 mM EDTA). Sonication was performed with Bioruptor (Diagenode). Lysates from 6 × 10^6^ cells were processed with the iDEAL ChIP kit (Diagenode) according to the manufacturer’s recommendations using 5 μg of antibody. Quantitative PCRs were performed using 2 μl of DNA in duplicate and enrichment was calculated related to input or Histone H3 values. Antibodies used: Histone H3 (ab1791, Abcam), H3K4me2 (07–030, Millipore) and H3K9me2 (39753, ActiveMotif).

### Analysis of promoter sequences

For each gene set, sequences from −150 to +50 bp relative to TSS were extracted using R biomaRt and BSgenome packages (hg19 genome assembly). Similarly, a full genome set of promoter sequences was built from the 23368 genes available from our RNA-seq data. It was reduced by randomly selecting 3 out of 7 sequences resulting in 78557 sequences for 18975 genes resulting in a file size less than 20 Mb for oPOSSUM analysis. For all of the gene sets, 4 to 7 promoter sequences per gene were obtained as a mean. oPOSSUM 3.0 was used for detecting the over-representation of transcription factor binding sites (TFBS) within a set of sequences as compared to a pre-compiled background set matching the GC composition of the target sequences. Position-weight matrices for NRF1 and NFY were downloaded from the database HomerMotifBD related to the HOMER suite (motif193 for NRF1 and motif181 for NFY) or JASPAR database for NRF1 motif in Supplementary Fig. S1a. Over-representation was assessed using Z-score (>10).

### Statistical analyses

For bioinformatic analyses, the number of TSS sequences containing at least one TFBS was compared between gene sets using a Fisher exact test. For all experimental work, statistical analyses were performed with Student t-test.

### Data availability

All data generated or analysed during this study are included in this published article and its Supplementary Information files.

## Electronic supplementary material

Supplementary data
