# Supplementary material for: LSD1-ERRα complex requires NRF1 to positively regulate transcription and cell invasion
Source: Sci Rep. 2018 Jul 3;8:10041. doi: 10.1038/s41598-018-27676-8 (PMC6030097; doi:10.1038/s41598-018-27676-8)
Supplement: Supplementary file 1 — Supplementary data [file 41598_2018_27676_MOESM1_ESM.pdf]

Supplementary File for

LSD1-ERRα complex requires NRF1 to positively regulate transcription and cell invasion

Ling Zhang, Julie Carnesecchi, Catherine Cerutti, Violaine Tribollet, Christelle Forcet, Séverine Périan, Jiemin Wong, and Jean-Marc Vanacker

Contains Supplementary Figures S1, S2, S3 and Supplementary Table S1.

Supplementary Figure S1. Additional bio-informatics data.
a. Same as Fig.1b using the displaying the percent of sequences displaying at least 85% similarity to the indicated NRF1 matrix. Except where indicated, significance is calculated relative to “all TSS” with ***: p<0.005, ns: nonsignificant.
b. Identification of the best (according to % score) putative NRF1 response elements (NRFRE) in the promoters (-150; +50 relative to TSS) of the ten genes of interest used in this study. Sequence, position on chromosome and relative to TSS is indicated.

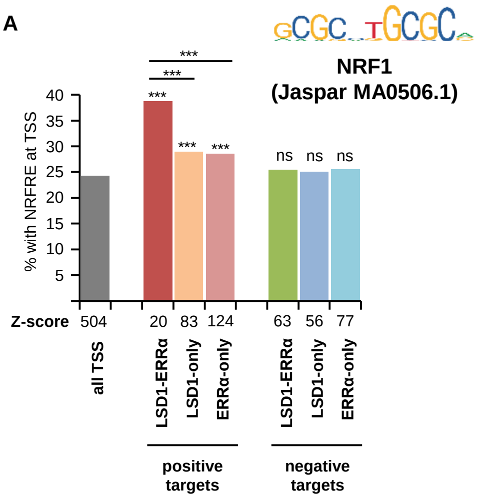

B

Gene ID	Chr.	NRFRE first nucleotide position: on chromosome	NRFRE first nucleotide position: rel. to TSS	strand	% score	NRFRE sequence
**ONECUT2**	18	55102819	-98	+	91.8	CCGCACGCGCA
**ZNF768**	16	30535412	-42	+	84.6	GCCCAGGCCCC
**GRWD1**	19	48949286	37	-	88.5	ACACGTGCGCC
**RPIA**	2	88991018	-144	+	87.2	GCGATTGCGCA
**RSAD1**	17	48556174	13	+	92.2	CCGGCTGCGCA
**ANKRD13B**	17	27916641	-146	+	90	CCCCCTGCGCG
**LEF1**	4	109002670	-70	+	87.1	GCAGAGGCGCA
**TMEM198**	2	220408794	32	-	87.8	GAGCCCGCCCG
**BSN**	3	49591895	-17	+	99.6	GCGCATGCGCA
**OXNAD1**	3	16306683	-13	-	87.8	GCGCGGGCGAG

Supplementary Figure S2. LSD1 requires both NRF1 and ERRα to activate common target genes. Same as Fig. 4b, examining the expression of the indicated target genes.

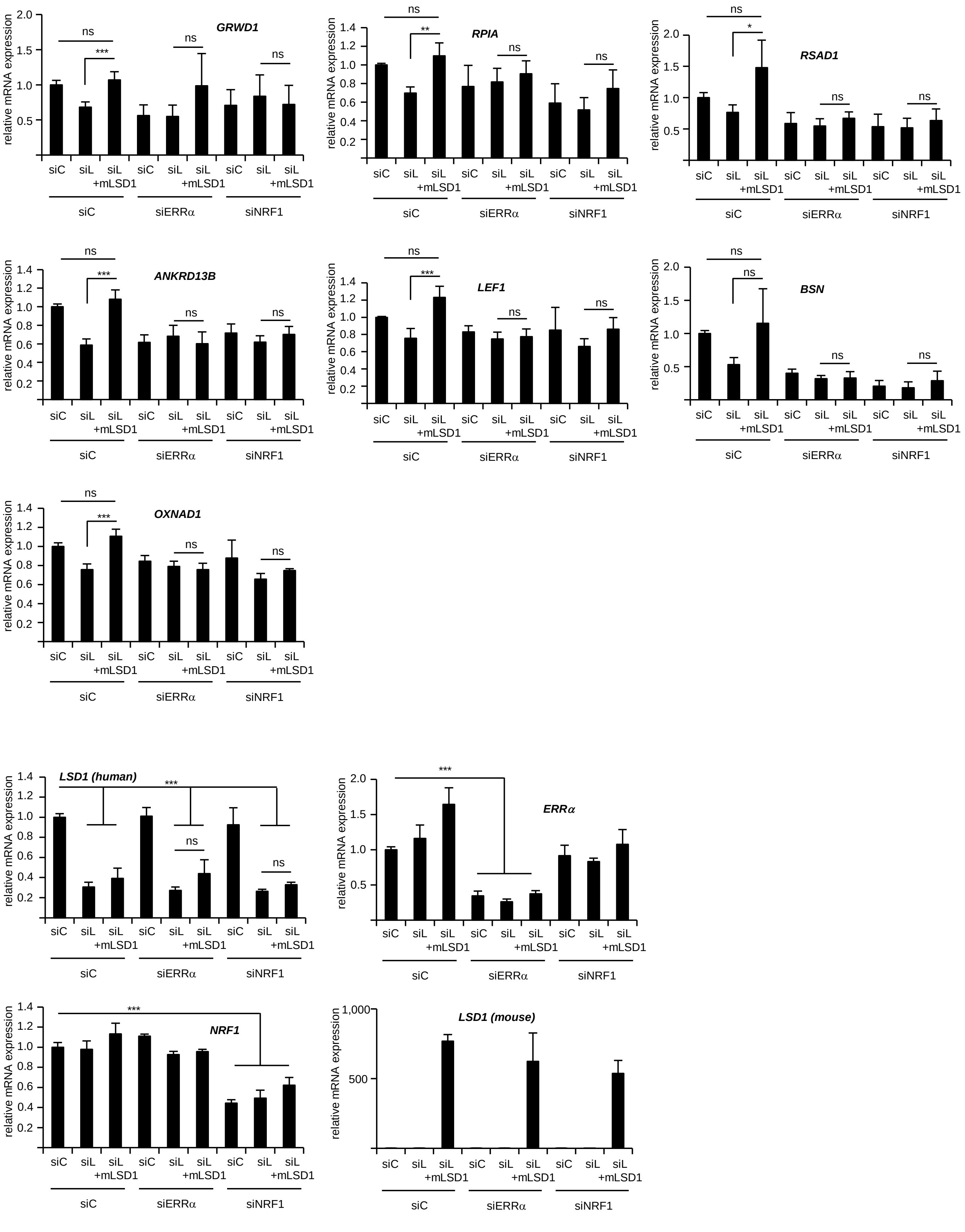

Supplementary Figure S3. Uncropped blots used for this study.

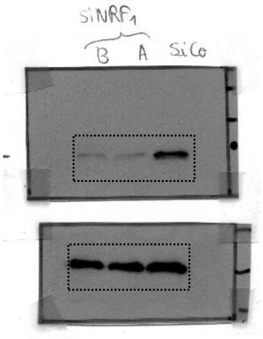

For Fig. 2A.
Note that the blots were left-right inverted in Fig. 2A.

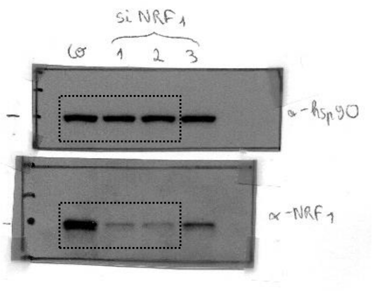

For Fig. 5B.
NRF1 and hsp90 parts

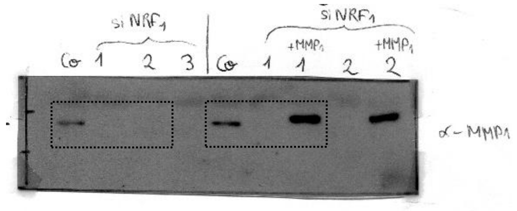

For Fig. 5B and 5E
MMP1 parts

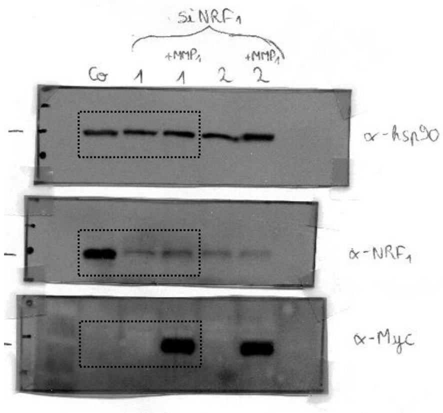

For Fig. 5E
Myc, NRF1 and Hsp90 parts

Supplementary Table S1: Oligonucleotides and siRNAs used in this study

Oligonucleotides for expression studies

36b4	GTCACTGTGCCAGCCCAGAA	TCAATGGTGCCCCTGGAGAT
KDM1A (LSD1) human	ACCACAACAGACCCAGAAGG	CTCGGTGGACAAGCACAGTA
ESRRA	CAAGCGCCTCTGCCTGGTCT	ACTCGATGCTCCCCTGGATG
NRF1	GCACCTTTGGAGAATGTGGT	AATTCCGTCGATGGTGAGAG
ONECUT2	AAATCTGGCAGGGAGACCTT	GGTTCTTGCTCTTTGCGTTT
ZNF768	GGGTACCTCAGAGGCAACAT	GGGTTCAAACTCTGGGCTTT
GRWD1	TGGTCACCGACTGAGAACAC	GCTCCAGCTGATGACATTGA
RPIA	TAGTCGCTTCATCGTGATCG	GATTCCCTTGTGCCACTGAT
RSAD1	CGCAGCTGAGATGTACCAGA	AACGCCAAGGTACTGACCAC
ANKRD13B	GGCAAGGTCAAAGGCTGTAA	GTGATCAGGGTCCCATTTTG
LEF1	TGGAAAACGAAGCTCATTCC	GGGTTGGCAGTGATTGTCTT
TMEM198	GTGCTGTTTGTTTGGAGTCG	TCTCGGTAGCAGAGGAGGAA
BSN	CCAGCCAAACTTCAACACCT	AGCCCTCTGCATCTGACAGT
OXNAD1	CAGCAGCTAAGGTGTGTGGA	AACCCACCAACCACAGAGAC
ABCF1	CAGTGCCAACCAGTGATGAG	GGCAGGCTTAGGAGGATGTT
SNX12	CCGAGGAGATGAAGGGATCT	TGTAGGCAGCGTTCATTCTG
ELK3	CCAAAGGCTTGGAAATCTCA	CGGAGTCAGAAGCAATCCAT
DARS	CCTGAGGCAGAAGGAGAAGA	GACTGGAGACGGAAGACTGC
CFDP1	GTTCCTTCAGCTCTGCCATC	TGAAGCTCTCCCAGTCCAGT
ASB13	GGAGCATCTGGACTGTGTCA	GTGGTGAAGGGCAGTCTCAT
TM9SF4	CCAGAACGATCCCGTAGAAA	TCTCAGCACCTCTCCCAGAT
NDUFB2	ACTGCTGGAGATGGTGGAGT	CACATGAGTCCGCTGAAGAA
MYC	GCCACGTCTCCACACATCAG	TCTTGGCAGCAGGATAGTCCTT
MMP1	AGGTCTCTGAGGGTCAAGCA	CTGGTTGAAAAGCATGAGCA
ACLY	TGCCGACTACATCTGCAAAG	GGTTCAGCAAGGTCAGCTTC
NEK7	TGGATGAGCAATCACAAGGA	TAGCCCATATCCGGTCGTAA
UHMK1	TGGCCTGCAGAGTGATACAG	AGCAGAACTGTTTGCCTTCC
Kdm1a mouse	CGAATGACCTCTCAGGAAGC	CCAGCCATAACTGCAATGTG

Oligonucleotides for ChIP on TSS

ONECUT2	GCCCTGATGGACTGAATGAA	GTGCCCAGACTTTCCATTGT
ZNF768	TTCTTCCTCAGGCTTTGGAG	CAGCAACAGCAAAGCACTTC
GRWD1	GATTAGCGGTCCCAGGAGTT	CTGCTGACACCCGGTAAGAG
RPIA	CGGAGAGCATTATGGGATTG	GGGTCTTGCTGGGAAATGTA
RSAD1	AGTTCTGAGCCAGGCACATC	CCTCCTGTCGGTTCTTTCC
ANKRD13B	TCCTCTCTCCCCATCATCC	GGTCCCCACAGCTGCATA
LEF1	ACACACCCCAAAACCAAGAC	GAAGGAGGTGGTGATTGAGG
TMEM198	GGGTTGGCAGTGATTGTCTT	TCTGCTCCTGACGTCACTC
BSN	CAGAGGCCGCTTCTAGTGG	CGCGGTTGTATCATGCTG
OXNAD1	TGGAGATGGCTGAACTAGGG	GCGAGCCCCACATTCTATT
ABCF1	AGGCTTCCCAGACAGTCGTA	GAGCTTCGCCGTCATCTC
SNX12	TGTGCTGCTAGGCAGGGTAT	GAGTGGAGGCCATATTCAGG
ELK3	GGCGGAAAAGCCTGTTTA	GAGCAGGAAGTTGGAGCAGT
MMP1	AGGAATCCATAAGGGGAGGA	CGGTGTCACCAGTGCTATCT

siRNAs

ERRα#1	GGCAGAAACCUAUCUCAGGUU	CCUGAGAUAGGUUUCUGCCUC
ERRα#2	GAAUGCACUGGUGUCACAUCUGCUG	CAGCAGAUGAGACACCAGUGCUUC
LSD1#1	ACUUUGUAACUGUCGAGCUGC	GCAGCUCGACAGUUACAAAGU
LSD1#2	CCACGGAGCGACAGAGCGAGC	GCUCGCUCUGUCGCUCCGUGG
NRF1#1	CAGUCACUAUGGCGCUUAA	UUAAGCGCCAUAGUGACUG
NRF1#2	CACGUUUGCUUCGGAAACU	AGUUUCCGAAGCAAACGUG
